# Sample Preparation as a Critical Aspect of Blood Platelet Mitochondrial Respiration Measurements—The Impact of Platelet Activation on Mitochondrial Respiration

**DOI:** 10.3390/ijms22179332

**Published:** 2021-08-28

**Authors:** Karolina Siewiera, Magdalena Labieniec-Watala, Nina Wolska, Hassan Kassassir, Cezary Watala

**Affiliations:** 1 Department of Haemostatic Disorders, Chair of Biomedical Sciences, Medical University of Lodz, Mazowiecka 6/8, 92-215 Lodz, Poland; n.m.wolska@gmail.com (N.W.); hassan.kassassir1@gmail.com (H.K.); cezary.watala@umed.lodz.pl (C.W.); 2 Department of Medical Biophysics, University of Lodz, Pomorska 141/143, 90-236 Lodz, Poland; magdalena.labieniec@biol.uni.lodz.pl; 3 Institute of Medical Biology, Polish Academy of Sciences, Lodowa 106, 93-232 Lodz, Poland

**Keywords:** blood platelets, mitochondria, mitochondrial respiration, platelet activation, sample preparation

## Abstract

Blood platelets are considered as promising candidates as easily-accessible biomarkers of mitochondrial functioning. However, their high sensitivity to various stimulus types may potentially affect mitochondrial respiration and lead to artefactual outcomes. Therefore, it is crucial to identify the factors associated with platelet preparation that may lead to changes in mitochondrial respiration. A combination of flow cytometry and advanced respirometry was used to examine the effect of blood anticoagulants, the media used to suspend isolated platelets, respiration buffers, storage time and ADP stimulation on platelet activation and platelet mitochondria respiration. Our results clearly show that all the mentioned factors can affect platelet mitochondrial respiration. Briefly, (i) the use of EDTA as anticoagulant led to a significant increase in the dissipative component of respiration (LEAK), (ii) the use of plasma for the suspension of isolated platelets with MiR05 as a respiration buffer allows high electron transfer capacity and low platelet activation, and (iii) ADP stimulation increases physiological coupling respiration (ROUTINE). Significant associations were observed between platelet activation markers and mitochondrial respiration at different preparation steps; however, the fact that these relationships were not always apparent suggests that the method of platelet preparation may have a greater impact on mitochondrial respiration than the platelet activation itself.

## 1. Introduction

Recently, there has been growing interest in blood platelets as mitochondria-related biomarkers of pathological changes in various diseases, including Alzheimer’s disease [1], sickle cell disease [2], sepsis [3], asthma [4] and diabetes [5]. Some of these reports suggest that the intensity of these changes could be a potential marker of clinical severity of disease [6], or even a prognostic factor for patient survival [7]. The use of platelets as biomarkers has a number of advantages: most importantly, sample collection is simple, routine, and relatively painless. In addition, platelet isolation from whole blood is quick, cheap, and does not require any specialized laboratory equipment; it has also been optimized over the years to obtain biological material devoid of other morphotic elements. However, despite the fact that platelets seem to be an excellent tool for studying mitochondrial-related diseases, their use can be complicated, mainly due to the risk of artefactual activation of these cells during sample handling and isolation. 

The activation of blood platelets leads to dramatic changes in their physiology and morphology. During this process, platelets secrete numerous bioactive substances from their intracellular granules and can release microparticles, which may contain functional mitochondria [8,9]. Therefore, it is crucial that samples are prepared with care and treated gently to avoid artifacts caused by *ex vivo*/*in vitro* platelet activation, which can potentially mask their true biological functioning. The fact that blood platelets are sensitive to activating stimuli, and more so in some pathophysiological states, may often present some methodological difficulties. The use of an inappropriate anticoagulant [10], centrifugation parameters [11], or even temperature [12] have been shown to be important factors that can lead to unintentional and undesirable platelet activation. Since the activation of platelets depends on the energy produced in the process of oxidative phosphorylation [13,14,15], changes in activation may also lead to alterations in mitochondria respiration, such as an increase in routine cell respiration related to elevated energy demands or a decrease in mitochondrial respiration related to the loss of mitochondria in the process of microparticle release. Unfortunately, this aspect is often overlooked or even ignored in the context of mitochondrial respiration measurement of platelets. To gain a greater understanding of the potential of platelets as biomarkers of mitochondrial functioning, the present study assesses whether the manner of the preparation of samples for respirometry measurements may lead to artefactual changes in platelet mitochondrial respiration; it also examines whether these artefactual changes are related to platelet activation and how much the results obtained with the use of some compared methods/variants differ from each other when used for the same biological material originating from a single subject.

Therefore, the aim of the present study was to compare different protocols of sample preparation for respirometric measurements and to verify which of them may induce changes in blood platelet mitochondrial respiration due to artefactual platelet activation. Elements that, in our opinion, may have an effect on *in vitro* platelet activation, such as the type of anticoagulant, the type of environment in which the platelets were suspended after isolation, the kind of respiration buffer, as well as the storage time before measurement, were taken into account when comparing different protocols of sample handling. The study also considered the changes in mitochondrial respiration parameters that can occur in response to direct platelet stimulation. For this purpose, increasing concentrations of ADP were used. Finally, we examined the associations between mitochondrial respiration parameters and platelet activation markers.

## 2. Results

### 2.1. The Impact of Anticoagulants on Platelet Activation and Mitochondrial Respiration

**Figure 1:**
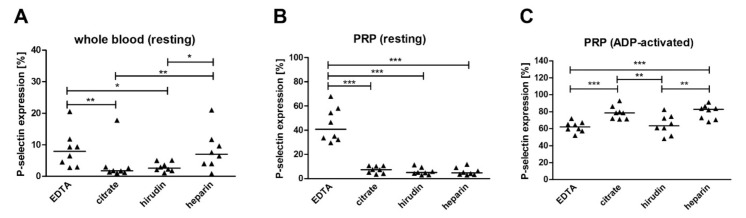
Activation and reactivity of platelets obtained from blood collected on different anticoagulants. Data presented as individual data points with medians (horizontal lines) (*n* = 8) for P-selectin expression in (**A**) resting platelets in whole blood, (**B**) resting platelets in PRP and (**C**) ADP-activated platelets in PRP. The significance of differences, as estimated with the bootstrap-boosted Kruskal–Wallis test (10,000 iterations) and the *post hoc* Conover–Inman test for multivariate comparisons, was: * *p* < 0.05, ** *p* < 0.01, *** *p* < 0.001.

The obtained data showed that ethylenediaminetetraacetic acid (EDTA) and unfractionated heparin did not protect blood platelets against undesirable activation during blood collection (Figure 1A). In addition, in the case of EDTA, the platelets were further activated during centrifugation of blood to platelet-rich plasma (PRP), which was not observed in the case of other anticoagulants (Figure 1B). At this stage, platelets in PRP obtained from blood collected on EDTA were activated to such an extent that their natural ability to respond to the stimulating factors, such as ADP, was inhibited. Such a problem was not observed for sodium citrate, hirudin or heparin (Figure 1C).

We have also observed the impact of EDTA on platelet mitochondrial respiration. ROUTINE respiration (*R*), a physiological coupling state controlled, among others, by the cellular energy demand, as well as the LEAK respiration (*L*), which is related to compensating for electron leak, proton leak, proton slip and cation cycling, was elevated when EDTA was used compared to other anticoagulants. The maximal electron transfer system capacity (ET capacity) did not differ between tested anticoagulants, but *R/E* and *L/E* ratios were higher for EDTA compared to other anticoagulants. The netROUTINE ratio, which represents the fraction of ET capacity used for the oxidative phosphorylation, remained unchanged, regardless of the tested variant. This indicates that enhanced ROUTINE respiration for EDTA samples was the result of an increased uncoupling/dyscoupling of respiration, and it was not related to elevated energy demands (Table 1).

The analysis showed that P-selectin expression was positively associated with ROUTINE respiration (both overall and after adjusting for the type of anticoagulant), as well as with LEAK respiration (overall) (Table 2 or Figure S1 in Supplementary Materials). A positive correlation was also noted between P-selectin expression and ET capacity when blood was collected using citrate or hirudin. Interestingly, when only EDTA was taken into account, the association between activation and respiration in the ROUTINE and LEAK states was negative (Table 2 or Figure S1). 

### 2.2. The Impact of Media and Respiration Buffers on Platelet Activation and Mitochondrial Respiration

**Figure 2:**
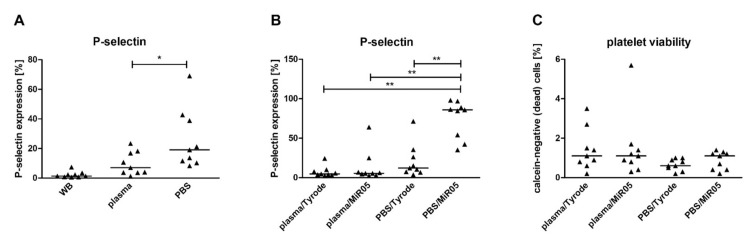
The influence of the method of sample preparation on platelet activation and viability. Data presented as individual data points with medians (horizontal lines); *n* = 9. (**A**) P-selectin’s presence measured on platelets in whole blood (WB) and on platelets isolated from that blood and suspended in plasma or in PBS (primary platelet suspensions); (**B**) P-selectin expression on isolated platelets suspended in different medium/buffer combinations; (**C**) viability of isolated platelets suspended in different medium/buffer combinations. Significance of differences was estimated with ANOVA for repeated measures with medium type as independent grouping variable (**A**) or with two-way ANOVA for repeated measures with the Tukey’s *post hoc* test (**B**,**C**): * *p* < 0.05, ** *p* < 0.001. Additional information on the effect of the medium, the effect of the buffer and the effect of the buffer depending on the medium are presented below: (**A**) *p* < 0.001 for the effect of isolation (WB vs. platelets resuspended in medium, plasma and PBS), (**B**) *p* < 0.001 for the effect of medium, *p* < 0.01 for the effect of buffer, *p* < 0.001 for the medium-dependent buffer effect; *p* < 0.05, plasma vs. PBS, *p* < 0.001, plasma vs. PBS/MiR05, PBS vs. PBS/MiR05, (**C**) ns. for the effect of medium, ns. for the effect of buffer.

Process of blood platelet isolation led to increased platelet activation; however, P-selectin expression was greater when after centrifugation cells were resuspended in PBS instead of plasma (Figure 2A). Similarly, when transferred to the respiration buffer, platelets suspended in phosphate buffered saline (PBS) were more prone to further activation than those suspended in plasma. Interestingly, the buffer most commonly used in respirometric measurements, MiR05, significantly activated platelets (Figure 2B). However, undesirable platelet activation did not occur when the platelet pellet was resuspended in autologous plasma, despite the fact that its final concentration in the respiratory buffer was only 5%. Regardless of the medium/buffer variant, no differences in platelet viability were observed (Figure 2C).

Blood platelet respiration was evaluated for final samples of platelets suspended in respiration buffers (medium/buffer variants). No significant differences in mitochondrial and non-mitochondrial respiration parameters were found between platelets resuspended in autologous plasma and platelets resuspended in PBS; however, such differences were revealed for respiration buffers. Increased respiration in the LEAK state and elevated ET capacity were observed when platelets were measured in MiR05 buffer (Table 3, the effect of buffer). The statistically significant differences between the four tested combinations were observed for platelets in plasma in relation to LEAK, ET capacity and ROX, for which the highest values were measured in MiR05 and between PBS/MiR05 and plasma/Tyrode for ET capacity (Table 3). 

Interestingly, we observed that no difference in electron transfer system (ETS) capacity was observed between plasma/MiR05 and PBS/MiR05, although the difference in the expression of P-selectin between these two variants reached 80%. On the other hand, despite the lack of statistically significant differences in platelet activation between plasma/MiR05 and plasma/Tyrode, significant differences in ET capacity between these variants were observed. This indicates that the method of preparation of the isolated platelets for oxygraphic measurement may have a greater impact on platelet respiration than the platelet activation itself in the course of this process.

### 2.3. The Impact of Storage Time on Platelet Viability, Artefactual Activation and Mitochondrial Respiration

**Figure 3:**
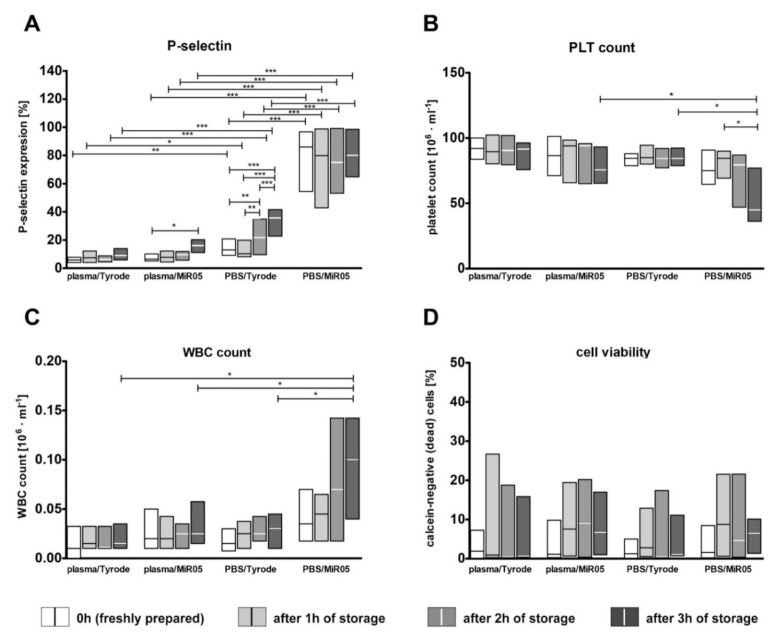
The effect of the storage of platelet suspension on platelet activation, platelet count and viability in samples suspended in either plasma or PBS and transferred to either MiR05 or Tyrode’s buffer at the appropriate time point of storage. Data are presented as medians (solid line within boxes) and IQRs (boxes); *n* = 6. P-selectin expression (**A**), platelet count (**B**), white blood cells count (**C**) and cell viability (**D**) were measured for freshly prepared platelet suspensions and after one, two and three hours of storage. The significances of the differences, as estimated with two-way ANOVA for repeated measures with Tukey’s *post hoc* test, were: * *p* < 0.05, ** *p* < 0.01, *** *p* < 0.001. Additional information about the effect of time, the effect of medium, the effect buffer is presented below: (**A**) *p* < 0.01 for the effect of buffer, *p* < 0.001 for the effect of time, *p* < 0.001 for the effect of medium; (**B**) *p* < 0.05 for the effect of time, *p* < 0.01 for the effect of medium, ns. for the effect of buffer; (**C**) *p* < 0.05 for the effect of medium, *p* < 0.01 for the effect of buffer, ns. for the effect of time; (**D**) ns. for the effect of time, ns. for the effect of medium, ns. for the effect of buffer.

Flow cytometric analysis showed that from the moment of preparation (0 h), platelets suspended in PBS were characterized by much higher activation than those suspended in plasma. In contrast, the initial activation of platelets suspended in plasma was lower than in PBS, but it increased during storage time. After three hours, the platelets suspended in plasma were significantly activated compared to freshly prepared ones (Table S2 in Supplementary Materials). However, neither variant demonstrated any effect of time on platelet viability or platelet count (Table S2). Platelet activation became even more noticeable after transferring platelets to respiratory buffers (Figure 3A). Statistical analysis revealed that the activation of blood platelets in samples prepared for oxygraphic measurement depended on time, medium and respiratory buffer. The highest activation was observed for platelets suspended in the PBS/MiR05 combination, and the smallest was observed for platelets suspended in plasma. No statistically significant differences in platelet activation were observed between the plasma/Tyrod and plasma/MiR05 setups (Figure 3A).

Interestingly, for the final suspensions, the number of platelets recorded by a hematological analyzer differed depending on the medium and the time point (Figure 3B). This was especially noticeable for platelets suspended in PBS/MiR05 compared to those suspended in PBS/Tyrode—both were prepared from the same platelet stock in PBS (Figure 3B). This difference was attributed to the underestimated values of platelet counts due to erroneous counting of the objects of ‘aggregating platelets’ as the objects of ‘white blood cells’ in the sample (Figure 3C). Therefore, when using such types of samples, standardization of the sample or normalization of the results should be based on values received for the primary suspension and not for that in the respiration buffer. Interestingly, platelet viability did not change during storage for up to three hours, regardless of the tested types of media (Figure 3D). 

**Figure 4:**
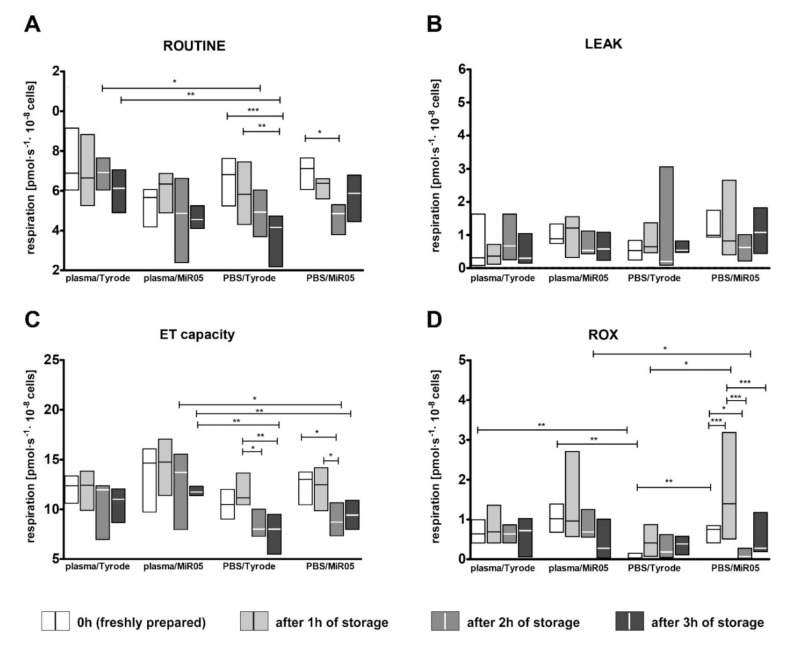
The effect of the storage of the platelet suspension on platelet mitochondrial and non-mitochondrial respiration in samples suspended in plasma or PBS and transferred to MiR05 or Tyrode’s buffer at the appropriate time point of storage. Data are presented as medians (solid line within boxes) and IQRs (boxes); *n* = 6. ROUTINE respiration (**A**), LEAK (**B**), ET capacity (**C**), and ROX (**D**) were measured for freshly prepared platelet suspensions and after one, two, and three hours of storage. The significance of differences, as estimated with two-way ANOVA for repeated measures with *post hoc* Tuckey test was: * *p* < 0.05, ** *p* < 0.01, *** *p* < 0.001. Additional information on the effect of time, the effect of medium and the effect of buffer are presented below: (**A**) *p* < 0.01 for the effect of time, *p* < 0.01 for the medium-depended buffer effect, ns. for the effect of medium, ns. for the effect of buffer; (**B**) ns. for the effect of time, ns. for the effect of medium, ns. for the effect of buffer; (**C**) *p* < 0.05 for the effect of medium, *p* < 0.001 for the effect of time, ns. for the effect of buffer; (**D**) *p* < 0.05 for the effect of time, *p* < 0.05 for the effect of medium *p* < 0.01 for the effect of time depending on medium ns. for the effect of buffer.

During the respirometric measurements, a decrease in ROUTINE respiration was observed over time for all the tested variants. However, it was more noticeable for platelets suspended in PBS than in plasma. No statistically significant decrease in respiration was observed after one-hour storage in relation to freshly-isolated platelets, regardless of the medium/buffer variant. Moreover, no statistically-significant changes in the all tested time points were observed for plasma/Tyrode and plasma/MiR05 (Figure 4A). Also, no differences in platelet activation were found between corresponding (matching) time points for these variants (Figure 3A). On the other hand, despite the colossal differences in platelet activation observed, among others between the same time point for plasma/MiR05 and PBS/MiR05, no changes in ROUTINE respiration were observed (Figure 4A).

Interestingly, storage time did not affect LEAK respiration, regardless of the medium used to prepare platelet suspension, the type of respiration buffer (Figure 4B) or even the activation of platelets over time observed for the PBS/Tyrode variant (Figure 3A). The ET capacity, on the other hand, decreased during the storage and this process depended on the medium in which the platelets were prepared (Figure 4C). However, no statistically significant changes were observed during the first hour after preparation, regardless of the medium/buffer variant. The decrease in ET capacity was observed after two hours of storage in the case of the PBS/MiR05 combination and after three hours in the case of the PBS/Tyrode variant (no changes for PBS/Tyrode 0 h vs. PBS/Tyrode 1 h, but a significant decrease between PBS/Tyrode 1 h and PBS/Tyrode 2 h). During this time, the P-selectin expression did not change in the case of PBS/MiR05 and increased by about 10% (up to 20%) in the case of PBS/Tyrode, indicating that the change in this parameter was not related with platelet activation. We also observed that there were also no significant differences for the tested time points between plasma/Tyrode and plasma/MiR05 in relation to ET capacity (Figure 4C). 

Interestingly, non-mitochondrial respiration (ROX) again seems to be higher when MiR05 was used as a respirometric buffer and when plasma was used as a medium (Figure 4D). Changes in the residual respiration of platelets in the medium/buffer variant of PBS/MiR05 turned out to be the least predictable over time of all the tested variants. This may be due to at least two mutually compounding factors: (1) PBS is only very weakly protective for platelets (compared to plasma), and (2) MiR05 exerts the highest platelet-activating effect.

### 2.4. The Impact of Platelet Activation on Mitochondrial Respiration

Respirometric measurements showed that after ADP addition, maximal respiration increased by 79%, 87% and 95%, respectively, for 1, 3 and 10 μM ADP, compared to the control, and the ROUTINE_ADP_ increased by between 30% and 38% compared to respiration before activation (Table 4). LEAK respiration did not change after platelet stimulation with ADP, while ET capacity was slightly, but significantly, lower in platelets stimulated with ADP in comparison with controls. Stimulation of platelets with ADP had no effect on non-mitochondrial oxygen consumption. The *L/E* control ratio did not change, thus confirming that changes in ET were negligibly small. The ROUTINE_ADP_*/E* and netROUTINE_ADP_ control ratios were 36–42% and 51–56% higher, respectively, in comparison to non-stimulated platelets. Additionally, the ratio of maximum oxygen consumption to ET capacity (MAX_ADP_*/E*) increased significantly following ADP addition (Table 4). Before ADP addition, ROUTINE respiration was the same for platelets in all of the measuring chambers, confirming the homogeneity of the measurement conditions (Table 4). Monitoring the activation markers confirmed increased platelet activation in samples stimulated with all ADP concentrations (Figure S2 Supplementary Materials).

**Figure 5:**
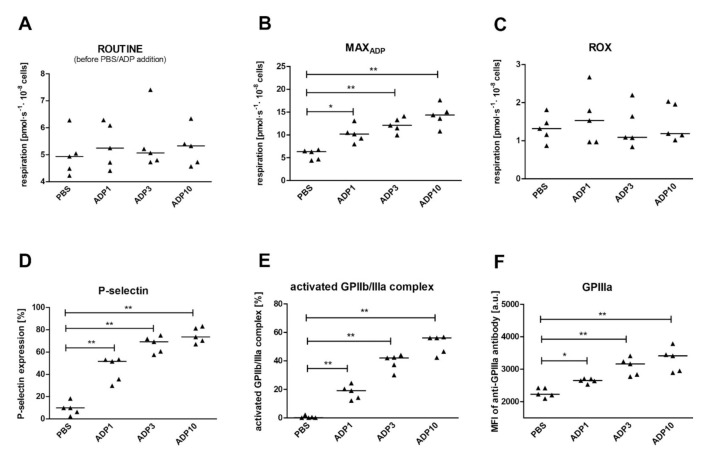
Oxygen consumption and cytometric markers of platelet activation in blood platelets stimulated with ADP. Data presented as individual data points with medians (horizontal lines); *n* = 5 for ROUTINE respiration before PBS or ADP addition measured in four different chambers of an oxygraph (**A**), maximal oxygen consumption after ADP addition (**B**), non-mitochondrial respiration (**C**), percentage of P-selectin-positive platelets (**D**), GPIIb/IIIa (activated complex)-positive platelets (**E**), and MFI of GPIIIa-bound antibodies (**F**) in resting (PBS) and ADP-activated platelets. Three ADP concentrations (1, 2, and 10 μM) were tested compared to the control. The significance of differences, as estimated with Friedman’s ANOVA (**A**) or ANOVA for repeated measures with Dunnett’s *post hoc* test (**B**–**F**), was: * *p* < 0.01, ** *p* < 0.001.

An additional experiment confirmed that the increase in mitochondrial respiration observed at the time of maximal oxygen consumption of ADP-activated platelets was not associated with a temporary increase in non-mitochondrial respiration (Figure 5C). Cytometric measurements of platelet surface antigens confirmed elevated platelet activation in response to stimulation (Figure 5D–F).

Moreover, the expression of platelet activation markers positively correlated with mitochondrial respiration in coupling states: MAX_ADP_ and ROUTINE_ADP,_ as well as their different ratios (Table 5 or Figure S3 in Supplementary Materials). However, the correlations were not always retained after adjustment for ADP concentration. The strongest positive correlations were observed between platelet activation parameters, and MAX_ADP_, (R_ADP_ − R)/R × 100) and MAX_ADP_/E. After adjustment for ADP concentration, the correlations were retained only between (R_ADP_ − R)/R × 100) and activated GPIIb/IIIa complex, and P-selectin. Interestingly, the strongest correlations with activation parameters were observed for activated GPIIb/IIIa complex, not for P-selectin (Table 5).

### 2.5. Predictors of Platelet Functioning during Activation: Multivariate Analyses

The canonical correlation coefficients between the common variables describing platelet activation and the common variables describing platelet mitochondrial respiration were evaluated (Table 6). The canonical variables, including the flow cytometric markers of platelet activation, demonstrated maximal values of extracted variances and significantly elevated extents of redundancy (considerable co-linearity and low tolerance). Canonical correlation coefficients were very high and significant in both of the separate sets of resting platelets or ADP-activated platelets, as well as for the overall set of data. An interesting observation was that the most significant contributors to the high canonical associations between both sets of variables appeared to be the P-selectin expression and the abundance of activated GPIIb/IIIa complex on the platelet surface. However, the fact that the extracted variance seldom exceeded 60% means that the ability of mitochondrial markers to explain platelet activation was only moderate. Another interesting observation was that the redundance for mitochondrial variables only seldom exceeded 30%, indicating that mitochondrial markers demonstrated low co-linearity and higher tolerance. The mitochondrial variables that highly contributed to the associations between the respiration of mitochondria and platelet activation were (R_ADP_ − R)/R × 100, MAX_ADP_ and ROUTINE_ADP_.

## 3. Discussion

Despite containing a small number of mitochondria, blood platelets seem to be promising candidates as easily accessible biomarkers of mitochondrial functioning under different pathological conditions [16]. However, platelets are highly sensitive to external stimuli, which can lead to artefactual activation [17] and can potentially affect mitochondrial respiration. Due to these limitations and the sensitive nature of the blood platelets, the present study examined how far the differentiated variants of procedures used for blood platelet preparation might affect platelet mitochondrial respiration. Our goal was to determine which of the different protocols of sample preparation is better and how much the results obtained with the use of some compared methods/variants differ from each other when used for the same biological material originating from a single subject.

### 3.1. Effect of Anticoagulants on Platelet Activation and Mitochondrial Respiration

Mitochondrial respiration measurements in autologous plasma seem to be very useful when environmental conditions must be as close as possible to the physiological state, and are often used to study various pathological conditions [7,18,19,20]. This approach minimizes the risk of artefactual activation of these cells during sample processing; however, it can also be more sensitive to the effect of the anticoagulants. Our results show that the use of EDTA not only led to a significant platelet activation, but also to increase in dissipative component of respiration, which is not accessible to perform biochemical work (LEAK). The EDTA is known for its negative effects on platelet activation [21,22], and despite this, it is still the most commonly used anticoagulant in platelet mitochondrial respiration studies [7,23,24,25,26,27]. While little research appears to have been performed on the effect of anticoagulants on platelet mitochondrial respiration, Kramer and colleagues reported that neither anticoagulant citrate-dextrose solution (ACD) nor EDTA had any effect on the bioenergetic function of platelets; however, no supporting data were given [28]. Therefore, to the best of our knowledge, this is the first report confirming that EDTA should not be used as an anticoagulant when evaluating platelet mitochondrial respiration, especially when the measurement is performed in plasma.

### 3.2. Effect of Media and Respiration Buffers on Platelet Activation and Mitochondrial Respiration

As oxygraphic measurements cannot be performed in whole blood, it is necessary to isolate platelets and suspend them in a new medium (usually in plasma or in PBS). Following this, in most cases, the cell suspension is transferred into buffer (typically MiR05), where the measurement is performed [23,27,29]. Our present findings indicate that platelets suspended in PBS were more activated than those suspended in plasma. Moreover, the buffer most commonly used in respirometric measurements, MiR05, significantly affects platelet functioning through their activation; however, this was avoided when plasma was used to suspend platelets after their isolation. Unlike MiR05, Tyrode’s buffer, one of the most frequently used buffers in the study of platelet function [30], did not cause any adverse changes in platelet activation, even when the platelets were suspended in PBS.

Moreover, our study revealed that the use of PBS instead of plasma did not affect mitochondrial respiration. Similar results were presented by Sjövall et al. [31] for platelet respiration tested directly in PBS or plasma, without the use of a respiration buffer. However, the final result of mitochondrial respiration in our study depended on the respiration buffer used. Platelets measured in MiR05 demonstrated an increase in LEAK respiration compared to Tyrode’s buffer. Interestingly, ET capacity was also much higher when platelets were measured in MiR05. This difference may be due to the fact that Tyrode’s buffer is not a typical respirometric buffer. The composition of such buffers seems to be less significant when measurements are performed on intact cells than isolated mitochondria [32]; however it can still be a possible cause of the present observations. Differences in LEAK and uncoupled state respiration have already been noted for isolated mitochondria measured in buffers with various levels of chloride ion concentration [32]. Therefore, although MiR05 can activate blood platelets and its use leads to elevated LEAK respiration, it is also associated with much higher ET capacity. Therefore, to keep platelets in as close to a physiological state as possible and yet still achieve the highest ET capacity values, a combination of plasma and MiR05 buffer should be used.

### 3.3. The Effect of the Short-Term Storage of Platelet Suspension on Platelet Activation and Mitochondrial Respiration

It is known that after isolation, platelets are more vulnerable to factors such as temperature, environmental changes, stimulating factors and time. During storage, they also undergo morphological and biochemical changes [33], and therefore our goal was to check for how long we can use the sample of platelets after their isolation. This is an important issue in respirometric measurements, since a limited number of samples can be tested simultaneously and the measurements are time consuming.

Our results show that blood platelets suspended in plasma demonstrated lower activation immediately after isolation and this increased with the storage time. Otherwise, the activation of platelets suspended in PBS was higher compared to that in plasma already after isolation, but it remained at a similar level over the tested time. This indicates that from the first steps of sample preparation, platelet functioning can be modified to a greater or lesser extent, and this can also affect the preservation of mitochondrial functioning over time. Our study showed that platelet storage led to a decrease in mitochondrial respiration in ROUTINE state, as well in the ET capacity in all the tested variants, but the biggest changes were noticed for platelets resuspended in PBS. When plasma was used, mitochondrial respiration was maintained at a similar level for up to three hours, regardless of which respiration buffer was further applied. The same direction of changes in mitochondrial respiration was observed by Villarroel et al. for platelet-rich plasma stored for up to 7 days [34]. Already at the first time point (24 h after isolation), ET capacity was reduced by 50% and decline in LEAK respiration was observed. A day later, a 75% decrease in ROUTINE respiration was recorded [34]. In turn, Sjövall et al. tested mitochondrial respiration after 48 h of storage of whole blood. They reported that the stored platelets demonstrated lower ET capacity than non-stored ones, as well as a decrease in oxidative phosphorylation [31]. Our results indicate that undesirable changes in platelet mitochondrial respiration may occur much earlier than after 24 h, and that the final effect depends both on the medium and the buffer used.

### 3.4. The Effect of Platelet Activation with ADP on Mitochondrial Respiration

In response to the stimulation of blood platelets with ADP, coupled physiological respiration increased to meet the requirements of elevated energy demands. A similar observation was made by Sowton et al. [35] after platelet stimulation with thrombin. Moreover, we noted a significant association between platelet activation and the mitochondrial parameters related to the elevated coupled respiration in response to ADP stimulation, especially when two higher concentrations were used. Among these parameters, the strongest associations occurred between variables describing platelet activation and: (a) maximal respiration after ADP addition; (b) the percentage increase in ROUTINE respiration in response to the stimulation (R_ADP_ − R)/R × 100); and (c) the ratio of maximal respiration to ET capacity. A strong positive correlation between platelet P-selectin expression and ROUTINE respiration was previously noted by our team in an animal model of diabetes [18]. Interestingly, we also observed a slight (4–7%) decrease in ET capacity for ADP-stimulated platelets in comparison to control. This potentially can be the result of the release of microparticles containing mitochondria [8,9], but that would also mean that the changes in coupled respiration were much greater.

These results clearly show the relationship between the changes in platelet functioning and mitochondrial respiration. Therefore, it is not surprising that undesirable platelet activation in the course of sample preparation may result in an alteration in mitochondrial respiration.

### 3.5. The Impact of Platelet Activation in the Course of Sample Preparation for Oxygraphic Measurements on Platelet Mitochondrial Respiration

During the evaluation of sample preparation elements which may affect platelet mitochondrial respiration, an increase in ROUTINE respiration was observed for platelets isolated from blood collected onto EDTA. These platelets were significantly activated compared to platelets isolated from blood collected using the other tested anticoagulants. However, in this case, the increase in ROUTINE respiration was in fact related to increase in the dissipative component of respiration (LEAK) and not with elevated energy demands, as shown by the unchanged netROUTINE parameter. Platelets activated with ADP, on the other hand, showed no increase in LEAK respiration. A potential explanation for this difference in LEAK respiration may be that the platelets in plasma demonstrate significantly higher mitochondrial respiration than those in the buffer [18]. In such a situation, changes that might remain unnoticed at a lower respiration rate can become significant when the rate is higher.

Interestingly, despite huge difference in the activation of platelets observed between some tested media/buffer variants, no differences in ROUTINE respiration, LEAK respiration and ET capacity were observed for these samples. Otherwise, despite of the lack of the differences in platelet activation between other samples (like plasma/MiR05 and plasma/Tyrode), significant differences in ET capacity were observed. In this case, the effect of buffer composition and not platelet activation was probably observed. A decrease in ET capacity that was not related to differences in respiration buffers, and could be the effect of platelet activation which was observed for PBS/MiR05 0 h vs. 2 h, and PBS/Tyrode 1 h vs. 3 h, when storage time was tested. This clearly shows that, despite the visible impact of platelet activation on mitochondrial respiration when samples are measured in the same conditions (such as in the case of the experiment with ADP stimulation), the effect of activation will not necessarily be observed when the samples prepared in different ways (e.g., different medium/buffer variants) are compared.

### 3.6. Study Limitations

In the course of this study, we did not evaluate the effects of sample preparation and platelet activation on individual mitochondrial complexes. We are not aware of the literature reports, which could indicate that platelet activation or applied sample preparation methods can lead to changes in the activities of individual mitochondrial complexes. Differences in respiration parameters presented in this study are, in our opinion, most probably related to the functioning of cells in various types of environment, which can further lead to differences in the functioning of membrane transporters or may impact cell energy demands. Noteworthily, the possible impact of platelet sample preparation and/or platelet activation on the functioning of individual complexes should certainly be assessed in the future. Nevertheless, we have to be aware that mitochondria respiratory measurements of such a type have their own requirements and may be influenced by other factors than those crucial for intact platelets. Due to this fact, the evaluation of the activities of individual mitochondrial complexes was not taken into consideration and not implemented as a part of this study.

## 4. Materials and Methods

### 4.1. Chemicals

The following anticoagulants were used for blood collection: sodium citrate (Becton-Dickinson, Franklin Lakes, NJ, USA), hirudin (Schering AG, Berlin, Germany), heparin (WZF Polfa S.A., Warsaw, Poland), and EDTA (Sarstedt, Warsaw, Poland). Antibodies against CD61, against CD62P and PAC-1 antibodies, mouse IgG_1_/PE isotype control, and isotype controls were from Becton Dickinson (Erembodegem, Belgium). Phosphate buffered saline (PBS) was obtained from Corning (New York, NY, USA). All material used for measurements and all other chemicals were obtained from Sigma (St. Louis, MO, USA), unless otherwise stated. Water intended to be used for the experiments (solution preparation, glassware washing) was previously purified using Easy Pure UF unit (Thermolyne Barnstead, Dubuque, IO, USA). 

### 4.2. Subjects

Human blood was obtained from healthy adult donors (seven men and 22 women; Caucasian; mean ages 29.4 ± 5.1 and 26.0 ± 6.7 years accordingly). All gave their written informed consent to participate. The exclusion criteria for the volunteers were as follows: age under 18 years, acute infection, chronic inflammatory disease including autoimmune disturbance(s), current cancer, current or previous blood diseases, arterial or venous thromboembolic events within the preceding six months, surgical procedures in the last six months, current anticoagulant therapy and the use of antiplatelet drugs. All participants testified that they had not been taking medications known to influence platelet function for at least 14 days prior to the study. The biochemical, morphological and medical characteristics of the participants of the study were presented in Table S1 (Supplementary Materials). Moreover, all experiments presented in this work were planned in a paired manner, which allowed us to minimize or eliminate the impact of the major confound factors.

The study was approved by the Ethics of Research in Human Experimentation Committee of the Medical University of Lodz (approval no. RNN/43/17/KE dated 14 February 2017). It was conducted in accordance with the guidelines established by the Declaration of Helsinki. 

### 4.3. Blood Collection and Sample Preparation 

Sampling of whole blood was performed in the morning, after overnight fasting and at least 15 min of resting. Blood was collected from the medial cubital vein in a laboratory tube with 3.2% sodium citrate, 25 ug/mL hirudin, 20 U/mL heparin or 1.6 mg/mL EDTA. Immediately after blood collection, the whole blood samples were used to evaluate platelet activation with the use of the flow cytometry technique. In the oxygraphic measurements, a suspension of platelets in autologous plasma or in the chosen buffer (Tyrode’s buffer or MiR05) was used. For this purpose, blood (supplemented with 62.5 ng/mL prostaglandin E_1_) was centrifuged (190 g with no break, 12 min, 37 °C) to obtain platelet-rich plasma. Following this, PRP was centrifuged (800 g with soft stop, 15 min, 37 °C, with the addition of 62.5 ng/mL prostaglandin E_1_) to isolate the blood platelets; the platelet pellet was suspended in either Tyrode’s buffer (134 mM NaCl, 0.34 mM Na_2_HPO_4_, 2.9 mM KCl, 12 mM NaHCO_3_, 1 mM MgCl_2_, 10 mM HEPES, pH 7.4, 5 mM glucose, 0.3% *w/v* bovine serum albumin), MiR05 buffer (110 mM sucrose, 60 mM K-lactobionate, 0.5 mM EGTA, 3 mM MgCl_2_, 20 mM taurine, 10 mM KH_2_PO_4_, 20 mM HEPES, pH 7.1 at 30 °C, and 0.1% *w/v* BSA essentially fatty acid free) or in plasma. Since the effect of platelet activation on mitochondrial respiration was tested, prostaglandin E_1_ was not added to the final platelet suspension after the centrifugation step. Platelet count and contamination with other cells were evaluated with the use of the Sysmex XS-800i hematology analyzer (Sysmex, Kobe, Japan). 

### 4.4. Flow Cytometric Analysis of Platelet Activation and Viability 

The activation of circulating platelets, as well as their *in vitro* response to stimulation with ADP (at final concentrations of 1, 3 and 10 µM) was evaluated according to Przygodzki et al. [36]. The expression of P-selectin and the active form of GPIIb/IIIa on GPIIIa-gated platelets were measured in whole blood immediately after blood collection, in PRP, in a suspension of platelets in plasma or PBS, or in platelets suspended in different medium/buffer combinations. The following parameters were recorded: the fractions of P-selectin-positive or the activated GPIIb/IIIa-positive platelets, and the mean fluorescence intensity (MFI) of GPIIIa. Ten thousand GPIIIa/PerCP-positive events were gathered using FACS Canto II flow cytometer (Becton-Dickinson, Franklin Lakes, NJ, USA).

Platelet viability was measured in resting platelets according to Rywaniak et al. [37]. A 1% solution of paraformaldehyde was used as a positive control (low viability). The percentage of calcein-negative signals within the population of ten thousand GPIIIa/PE-positive events (platelets) was measured using a FACS Canto II flow cytometer.

### 4.5. Measurement of Mitochondrial Respiratory Parameters 

Mitochondrial respiration was recorded *in vitro* by—a continuous measurement of oxygen consumption with a high-resolution respirometer equipped with a Clark-type polarographic oxygen sensor (Oxygraph-2k, Oroboros Instruments, Innsbruck, Austria). This sensor consists of a gold cathode, silver/silver chloride anode and electrolyte, which are separated from the analyte by the gas-permeable membrane. It allows to measure changes in the oxygen partial pressure of a solution. The applied voltage leads to the reduction of oxygen on the cathode, while silver is oxidized at the anode. This provides a current that is proportional to oxygen’s partial pressure in the experimental solution. Mitochondrial respiration is derived from the rate of oxygen concentration in a closed chamber, which decreases as the tested biological material consumes oxygen [38]. Therefore, mitochondrial respiration can be tested in real-time by applying appropriate substrate-uncoupler-inhibitor titration (SUIT) protocol during the experiment. 

Experiments were performed at 37 °C in the 2 mL chambers with the stirrer speed set to 750 rpm. Zero calibration and instrumental background calibration were performed according to the manufacturer’s instructions on a regular time basis. Calibration at air saturation in Easy Pure filtrated water (for measurements in plasma) or in respiration media (MiR05 or Tyrode’s buffer, depending on which was used for measurements) was conducted every day before an experiment started. The following solubility factors were set up for these calibrations and subsequent measurements: 1.0 for water, 0.89 for plasma, and 0.92 for MiR05 and Tyrode’s buffer [31]. Moreover, the chemical background of the added substances was also tested to ensure that there were no differences between the buffers in response to chemicals added to the chamber. Data were recorded using a dedicated software with recording interval of 2 s (DatLab software 7.0, Oroboros Instruments, Innsbruck, Austria).

To test mitochondrial respiration, platelets were resuspended in plasma, Tyrode’s buffer or MiR05 to obtain a concentration of 1 × 10^8^ platelets/mL. Two milliliters of such a suspension were added to each oxygraphic chamber. Measurements were performed according to Gnaiger and Renner-Sattler [39]. The following SUIT protocol was applied: first, the platelet oxygen consumption was allowed to stabilize, and the respiration was captured in a physiological coupling state (ROUTINE, *R*)*;* oligomycin was then added (0.25 µM in the case of measurements in plasma or 0.025 µM in the case of measurements in buffers) to evaluate the dissipative component of respiration (LEAK respiration, *L*); next, the maximum electron transfer capacity (ET capacity, *E*) was measured by titration with the 0.25 µM aliquots of carbonyl cyanide p-trifluoromethoxyphenylhydrazone (FCCP), until no further increase in respiration was observed; finally, rotenone (0.5 µM) and antimycin-A (2.5 µM) were added to evaluate the residual, non-mitochondrial oxygen consumption (ROX). The oxygen consumption was expressed as pmol O_2_ per second per 10^8^ cells (pmol × s^−1^ × 10^−8^ cells). All values recorded for the states of ROUTINE, LEAK and ET capacity were corrected for ROX.

In addition, to better assess the differences in a mitochondrial functioning between the tested variants, the following flux control ratios were calculated: ROUTINE control ratio (*R/E*) indicating the portion of the electron transfer (ET) capacity used during routine cell activity; LEAK control ratio (*L/E*) indicating the proportion of uncoupled or dyscoupled respiration to ET capacity; and netROUTINE control ratio ((*R − L)/E*), indicating the proportion of total electron transfer capacity used for oxidative phosphorylation [39].

### 4.6. Study Design

The aims of the present study were to (i) compare different protocols of sample preparation for respirometric measurements in order to identify which of these procedures may lead to “changed” blood platelet mitochondrial respiration, and (ii) assess whether the effect of sample preparation on mitochondrial respiration might be the result of platelet activation during sample handling. Therefore, we investigated the impact of several factors on blood platelet mitochondrial respiration and activation, including:

(a) Commonly used anticoagulants (EDTA, sodium citrate, hirudin, heparin): for this purpose, blood was collected using four different anticoagulants from each subject (*n* = 8). Experiments were conducted in intact platelets suspended in autologous plasma. As blood platelets in plasma appeared to be less sensitive to the uncoupler, for these measurements, FCCP was titrated in 2.25 µM aliquots.
(b) The type of medium in which platelets are resuspended after isolation to prepare stock of cells, and the type of respiration buffer in which platelets are suspended for the measurements: for this purpose, blood was collected using sodium citrate, centrifuged to PRP and the platelet concentration was assessed. Next, PRP was evenly divided into two tubes and centrifuged to obtain platelet sediment, which was suspended in a small volume of autologous plasma or PBS to obtain a concentration close to 20 × 10^8^ platelets/mL. The blood platelet concentration was validated again and the necessary number of platelets were transferred to the respiration buffer (Tyrode’s buffer with 0.3% of BSA or MiR05) to obtain 1 × 10^8^ platelets/mL. The influence of two factors (medium and buffer) was examined at the same time in the following combinations: plasma/Tyrode, plasma/MiR05, PBS/Tyrode, PBS/MiR05; all of these were prepared from the blood of one subject (*n* = 9). Additionally, changes in the viability of the blood platelets were assessed.
(c) The effect of storage time: we evaluated whether the results of platelet mitochondrial respiration will differ over time; measurements were taken every hour during three hours of storage from the sample preparation step. For this purpose, blood was collected using sodium citrate (*n* = 12). Isolated platelets were resuspended in autologous plasma or in PBS with 5 mM glucose and stored in the dark at 37 °C with gentle mixing by inversion every half an hour. At each time point, platelets were transferred to the respiration buffer (Tyrode’s buffer with 0.3% BSA or MIR05) to obtain 1 × 10^8^ platelets/mL. As the blood platelets appeared to be more sensitive to FCCP at the one- to three-hour time points, for these measurements, FCCP was added in 0.125 µM aliquots instead of 0.25 µM aliquots. Additionally, changes in the viability of platelets during storage were verified.
(d) The effect of platelet activation: for this purpose, blood was collected using sodium citrate (*n* = 10). Isolated platelets were suspended in plasma/MiR05, as in point c above. ROUTINE respiration was allowed to stabilize and then either ADP (1 µM, 3 µM, 10 µM, final concentrations) or PBS was added. After ADP addition to the oxygraphic chamber, an immediate increase in oxygen consumption was observed; this quickly reached the maximum rate (MAX_ADP_) and began to decrease. When respiration stabilized, ROUTINE respiration in ADP-activated platelets were measured (ROUTINE_ADP_) and the standard protocol was continued. Oxygen consumption in ROUTINE state, MAX_ADP_, ROUTINE_ADP_, LEAK state and for ET capacity was ROX-corrected. In addition, to better assess the differences in mitochondrial functioning between the tested variants, the following ratios were calculated: MAX_ADP_/*E*, *R_ADP_/E*, netROUTINE_ADP_ ((*R_ADP_ − L)/E*) and *(R_ADP_ − R)/R* × 100.

A supplementary experiment was performed to confirm that the increase in mitochondrial respiration at the time of maximal oxygen consumption in ADP-activated platelets was not associated with a temporary increase in non-mitochondrial respiration. For this purpose, ROX was assessed immediately after maximum respiration was obtained (*n* = 5).

### 4.7. Statistical Analysis

As a number of analyzed variables were not homogeneous with respect to normality and variance, all data were presented as median and interquartile range (IQR) (lower (25%) to upper (75%) quartile); this enabled us to easily compare numerical data between experiments and between related variables. The Shapiro–Wilk test was used to test for normality, Levene’s test was used to verify variance homogeneity, and Mauchly’s test was used to check for data sphericity. Where the data demonstrated a normal distribution and homoscedasticity/sphericity, parametric tests were used for further analyses; otherwise, non-parametric tests were employed. The statistical significance of differences between homogenous groups was tested using one-way ANOVA, one-way ANOVA for repeated measures or two-way ANOVA for repeated measures, followed by a *post hoc* Tukey’s test, the least significant difference (LSD) test or Dunnett’s test for multiple comparisons. The non-normally distributed variables and those demonstrating heterogeneous variances were tested using Friedman’s ANOVA followed by Dunn’s *post hoc* test, or the non-parametric Kruskal–Wallis test followed by the *post hoc* non-parametric Conover–Inman test. The sidedness of the tests was selected based on the null hypothesis, indicating the expected direction of an effect. 

The associations between variables were analyzed with Spearman’s rank correlation. Canonical analysis was performed to determine which of the studied variables contributed the most to the significant association between selected sets of parameters describing either the flow cytometric hallmarks of blood platelet activation (set 1) or the platelet mitochondrial respiration (set 2). This analysis enabled us to obtain a common (canonical) variable for each of these sets. The results indicate how various analyzed variables contributed to the formation of the strongest associations between blood platelet activation and platelet mitochondrial respiration. 

As a standard, due to relatively small sample sizes and in order to ensure the sufficient statistical power of estimated inferences and associations, we employed the technique of resampling bootstrap (with 5000 or 10,000 iterations) to indicate the likelihood that the revealed differences/associations could be observed by pure chance. In such situations, we refer in the description of the outcomes to the bootstrap-boosted test statistics. Statistical analyses were performed using Statistica v.13 (Dell Inc., Tulsa, OH, USA), Resampling Stats Add-In for Excel v.4 (The Institute for Statistics Education, An Elder Research Company, Arlington, VA, USA) and GraphPad Prism v.5 (GraphPad Software, San Diego, CA, USA).

## 5. Conclusions

Blood platelets are clearly very sensitive to even subtle changes in their microenvironment. This fact can have both positive and negative implications on their use as potential biomarkers. The disadvantageous aspect of this sensitivity is related to the difficulties in preparing the samples in a way that does not affect the tested parameters. Our results clearly indicate that the method of sample preparation can lead to changes in platelet mitochondrial respiration. These changes seem to be related to, but not limited to, platelet activation during the process. Moreover, the method of preparation of isolated platelets for oxygraphic measurements may have an even greater impact on mitochondrial respiration than the platelet activation during this process. Therefore, the choice of anticoagulant, medium, or respiration buffer, and the decision to limit the storage time of samples, or prevent platelet activation in general, seem to be key considerations in the use of platelets as a biomarker of mitochondrial changes. In addition, any comparison of outcomes between research groups using different methods of platelet preparations can be a difficult task and certainly needs to be carried out with an understanding of the discrepancies resulting from the different preparation methods and should take into account the extent of related platelet activation. Therefore, this paper can be treated not only as a mini-guide on the preparation of platelets used for respirometric measurements, but also as a source of knowledge about the differences between various methods of sample preparation for those researchers who want to reliably compare their results with those reported by different teams or for groups that consider the merging of their data. It can also act as a specific alert for researchers, warning them about the possibility of obscuring actual data by the modification of mitochondria functioning in the process of platelet preparation.

## Figures and Tables

**Table 1:** Mitochondrial respiration parameters and non-mitochondrial respiration of blood platelets in autologous plasma obtained from blood collected using various anticoagulants.

Anticoagulant	ROUTINE *R*	LEAK *L*	ET *E*	ROX	*R/E*	*L/E*	netROUTINE (*R − L*)/*E*
	(pmol × s^−1^ × 10^−8^ cells)				(a.u.)		
**citrate**	7.50; 6.62–7.86	0.34; 0.14–0.46	13.43; 12.33–18.30	3.35 2.61–3.92	0.51; 0.51–0.56	0.03; 0.00–0.03	0.50; 0.48–0.56
**hirudin**	6.14; 5.72–7.56	0.32; 0.23–0.46	13.97; 12.15–16.73	3.57; 3.36–5.06	0.48; 0.43–0.53	0.02; 0.01–0.04	0.43; 0.4–0.48
**heparin**	6.65; 5.98–7.48	0.36; 0.03–0.81	12.94; 12.09–16.03	3.98; 3.18–4.42	0.50; 0.42–0.54	0.03; 0.00–0.07	0.46; 0.42–0.50
**EDTA**	9.96; 8.4–16.60	2.20; 1.04–7.58	14.45; 13.25–17.67	2.95; 2.72–4.24	0.77; 0.59–0.85	0.19; 0.07–0.41	0.49; 0.47–0.65
Significance by inference tests	* p= 0.003 EDTA vs. citrate, hirudin, heparin ^#^*P*_1α_ < 0.03	* p = 0.013 EDTA vs. citrate ^#^ *p*_1α_ = 0.023 EDTA vs. hirudin ^#^ *p*_1α_ = 0.049 EDTA vs. heparin ^#^ *p*_1α_ = 0.034	ns.	ns.	* *p* = 0.014 EDTA vs. citrate ^#^ *p*_1α_ = 0.048 EDTA vs. hirudin ^#^ *p*_1α_ = 0.026 EDTA vs. heparin ^#^ *p*_1α_ = 0.028	* *p* = 0.020 EDTA vs. citrate ^#^ *p*_1α_ = 0.026 EDTA vs. hirudin ^#^ *p*_1α_ = 0.04 EDTA vs. heparin ^#^ *p*_1α_ = 0.075	ns.

Data presented as medians and IQR; *n* = 8. Two-sided (*p*) or one-sided (*p*_1α_) significance of differences between tested variants estimated with bootstrap-boosted Kruskal–Wallis test (10,000 iterations) (*) and the bootstrap-boosted Mann–Whitney U-test with Bonferroni’s correction for multiple comparisons (^#^); ns., not significant.

**Table 2:** Associations between mitochondrial respiration parameters and P-selectin expression and for blood platelets suspended in autologous plasma obtained from blood collected using various anticoagulants.

Anticoagulant	ROUTINE *R*	LEAK *L*	ET *E*	ROX	*R/E*	*L/E*	netROUTINE (*R − L*)/*E*
	Bootstrap-boosted Spearman R_S_ in groups, P_1-sided_						
**citrate**	−0.183, ns.	0.235, ns.	0.691, *p* < 0.0001	−0.838, *p* < 0.0001	−0.828, *p* < 0.0001	0.038, ns.	−0.826, *p* < 0.0001
**hirudin**	0.002, ns.	0.206, ns.	0.315, *p* = 0.040	0.316, *p* = 0.039	−0.144, ns.	0.191, ns.	−0.114 ns.
**heparin**	−0.194, ns.	−0.565, *p* = 0.0004	0.062, ns.	0.121, ns.	−0.051, ns.	−0.511, *p* = 0.001	0.259, ns.
**EDTA**	−0.339, *p* = 0.029	−0.407, *p* = 0.010	−0.046, ns.	−0.496, *p* = 0.002	−0.087, ns.	−0.332, *p* = 0.032	0.195, ns.
Overall^@^ and partial ^&^ (*in parentheses*) Spearman *R*_S,_ *P*_1-sided_	^@^ 0.428, *p* = 0.007 ^&^ (0.314, *p* = 0.040)	^@^ 0.321, *p* = 0.037 ^&^ (0.108, ns.)	^@^ 0.284, ns. ^&^ (0.273, ns.)	^@^ −0.207, ns. ^&^ (−0.237, ns.)	^@^ 0.264. ns. ^&^ (0.089, ns.)	^@^ 0.273. ns. ^&^ (0.048, ns.)	^@^ 0.020. ns. ^&^ (0.034, ns.)

Bootstrap-boosted simple Spearman correlation coefficients (Rs) and bootstrap-boosted partial Spearman correlation coefficients adjusted for anticoagulant type; *n* = 32 (5000 iterations). Bootstrap-boosted Spearman correlation coefficients between mitochondrial respiration variables and P-selectin expression in resting platelets in PRP are given as either the overall (^@^ *R*_S_) or the partial coefficients (^&^ *R*_S p,_ adjusted for the type of anticoagulant); ns., not significant.

**Table 3:** Mitochondrial respiration parameters measured for platelets resuspended in four different medium/buffer combinations.

Condition Medium/Buffer	ROUTINE *R*	LEAK *L*	ET *E*	ROX
	(pmol × s^−1^ × 10^−8^ cells)			
plasma/MiR05	5.05; 4.32–6.06	0.61; 0.28–1.16	13.17; 11.02–13.93	1.26; 0.50–3.43
plasma/Tyrode	6.07; 5.10–6.76	0.19; 0.09–0.73	9.95; 9.29–11.41	0.66; 0.25–1.04
PBS/MiR05	6.38; 5.30–7.12	0.77; 0.37–1.14	13.27; 10.64–15.14	1.27; 1.19–2.43
PBS/Tyrode	6.05; 3.92–7.79	0.29; 0.09–0.38	11.33; 9.15–13.96	0.68; 0.38–1.21
Statistical significance of the effect of medium: plasma vs. PBS	ns.	ns.	ns.	ns.
Statistical significance of the effect of buffer: MiR05 vs. Tyrode	ns.	*p* < 0.05	*p* < 0.05	*p* = 0.0706
Statistical significance of the differences between the four tested combinations		*p* < 0.05 plasma/MiR05 vs. plasma/Tyrode	*p* < 0.05 PBS/MiR05 vs. plasma/Tyrode, plasma/MiR05 vs. plasma/Tyrode	*p* < 0.05 plasma/MiR05 vs. plasma/Tyrode
Statistical significance according to bootstrap-boosted test		*p* < 0.05 plasma/MiR05 vs. plasma/Tyrode	*p* < 0.01 plasma/MiR05 vs. plasma/Tyrode, *p* < 0.001 PBS/MiR05 vs. plasma/Tyrode,	*p*< 0.05 plasma/MiR05 vs. plasma/Tyrode

Data presented as medians and IQR; *n* = 9. Significance of differences was estimated with ANOVA for repeated measures with the *post hoc* LSD test. Significance of differences was additionally confirmed with the bootstrap-boosted test adjusted for multiple comparisons (10,000 iterations); ns., not significant.

**Table 4:** Mitochondrial respiration parameters, control ratios and non-mitochondrial respiration of resting and ADP-stimulated platelets.

	ROUTINE *R*	MAX_ADP_	ROUTINE_ADP_ *R_ADP_*	LEAK *L*	ET *E*	ROX	*R/E*	*R*_ADP_/*E*	netROUTINE_ADP_ (*R*_ADP_ − *L*)/E	MAX_ADP_/*E*	(*R*_ADP_ − *R*)/R*100
	(pmol × s^−1^ × 10^−8^ cells)						(a.u.)				(%)
resting (PBS)	6.19; 4.86–6.87	6.80; 5.61–7.92	5.93; 5.26–7.13	0.69; 0.28–0.98	12.40; 11.01–14.00	1.11; 0.71–1.30	0.49; 0.40–0.53	0.47; 0.44–0.53	0.40; 0.36–0.50	0.50; 0.48–0.63	5.77; 8.15–13.20
ADP 1 μM	5.49; 4.86–6.74	12.18; 11.75–12.87	7.72; 6.70–8.18	0.37; 0.18–0.54	11.45; 10.24–12.77	1.20; 0.82–1.57	0.51; 0.43–0.56	0.64; 0.59–0.69	0.62; 0.54–0.67	1.03; 0.94–1.16	30.01; 17.91–39.13
ADP 3 μM	5.43; 5.04–5.94	12.72; 11.50–13.74	8.18; 6.77–8.38	0.27; 0.09–0.55	11.51; 10.66–12.80	1.33; 0.94–1.67	0.47; 0.43–0.54	0.66; 0.63–0.71	0.63; 0.60–0.66	1.07; 0.99–1.17	37.73; 30.69–53.52
ADP 10 μM	5.66; 5.09–6.26	13.29; 12.04–16.35	7.72; 7.17–8.40	0.31; 0.21–0.78	11.98; 10.42–13.01	1.30; 0.97–1.47	0.47; 0.45–0.55	0.68; 0.61–0.70	0.60; 0.57–0.66	1.10; 1.03–1.31	35.93; 30.57–41.14
Statistical significance	^#^ ns.	*^#^ p* < 0.001 PBS vs. ADP1, PBS vs. ADP3, PBS vs. ADP10	*^@^ p* < 0.05 PBS vs. ADP3, PBS vs. ADP10	*^#^* ns.	*^#^ p* < 0.05 PBS vs. ADP1, PBS vs. ADP3, PBS vs. ADP10	*^#^*ns.	*^#^* ns.	*^#^ p*< 0.001 PBS vs. ADP1, PBS vs. ADP3, PBS vs. ADP10	*^#^ p* < 0.001 PBS vs. ADP1, PBS vs. ADP3, PBS vs. ADP10	*^#^ p* < 0.001 PBS vs. ADP1, PBS vs. ADP3, PBS vs. ADP10	*^#^ p* < 0.001 PBS vs. ADP1, PBS vs. ADP3, PBS vs. ADP10
Statistical significance by bootstrap-boosted		*p* < 0.001 PBS *vs*. ADP1, PBS vs. ADP3, PBS vs. ADP10	*p* < 0.01 PBS vs. ADP1, PBS vs. ADP3, PBS vs. ADP10		*p* < 0.01 PBS vs. ADP3, PBS vs. ADP10			*p* = 0.001 PBS vs. ADP1; *p* < 0.001 PBS vs. ADP3, PBS vs. ADP10	*p* = 0.002 PBS vs. ADP1; *p* < 0.001 PBS vs. ADP3, PBS vs. ADP10	*p* < 0.001 PBS vs. ADP1, PBS vs. ADP3, PBS vs. ADP10	*p =* 0.002 PBS vs. ADP1; *p* < 0.001 PBS vs. ADP3, PBS vs. ADP10

Data presented as medians and IQR, *n* = 9. Three ADP concentrations (1, 3 and 10 μM) were tested compared to control (PBS). General significance of differences was estimated with either ANOVA for repeated measures (^#^) or Friedman’s test (^@^) with Dunnett’s *post hoc* test and the bootstrap-boosted Bonferroni-corrected paired Student *t* test for the *post hoc* multiple comparisons between control (PBS) and particular ADP concentrations; ns., not significant.

**Table 5:** Associations between mitochondrial parameters and variables describing blood platelet activation with ADP.

Variable	P-Selectin (%)	Activated GPIIb/IIIa Complex (%)	GPIIIa (a.u.)
MAX_ADP_	0.580, *p* < 0.0001 (−0.050, ns.)	0.624, *p* < 0.0001 (0.142, ns.)	0.587, *p* < 0.0001 (0.168, ns.)
ROUTINE_ADP_	0.402, *p* = 0.008 (0.172, ns.)	0.428, *p* = 0.005 (0.239, ns.)	0.372, *p* = 0.013 (0.170, ns)
ET capacity	−0.011, ns. (0.204, ns.)	0.024, ns. (0.225, ns.)	0.149, ns. (0.348, *p* = 0.019)
*R*_ADP_/*E*	0.358, *p* = 0.016 (−0.142, ns.)	0.391, *p* = 0.009 (−0.017, ns.)	0.297, *p* = 0.039 (−0.094, ns.)
netROUTINE_ADP_	0.320, *p* = 0.028 (−0.133, ns.)	0.403, *p* = 0.007 (0.072, ns.)	0.236, ns. (−0.137, ns.)
(*R*_ADP_ − *R*)/R*100	0.641, *p* < 0.0001 (0.294, *p* = 0.041)	0.734, *p* < 0.0001 (0.513, *p* = 0.001)	0.544, *p* < 0.0001 (0.208, ns.)
MAX_ADP_/*E*	0.503, *p* = 0.001 (−0.264, ns.)	0.579, *p* < 0.0001 (0.026, ns.)	0.522, *p* = 0.001 (0.040, ns.)

Bootstrap-boosted simple Spearman correlation coefficients (Rs), *P*_1-sided_ and bootstrap-boosted partial Spearman correlation coefficients adjusted for ADP concentration, *P*_1-sided_; *n* = 36 (5000 iterations); ns., not significant.

**Table 6:** Canonical correlations between two sets of parameters: flow cytometry markers of platelet activation and parameters of platelet mitochondrial respiration.

Resting/Activated Cells	Platelet Reactivity/Activation (Set 1)	Extracted Variance [%]	Total REDUNDANCE [%]	Explanatory Variables (Set 2)	Extracted Variance [%]	Total Redundance [%]	Canonical Correlation [R]	Canonical Determination [R^2^]	*p*	Wilks’ Lambda	Best Contributors
resting platelets	*cytometric*	100.0%	52.8%	*mitochondrial*	58.1%	38.2%	0.960	0.921	0.000	0.008	P-selectin, GPIIb/IIIa_ADP_, (*R*_ADP −_*R*)/*R* × 100, MAX_ADP_, ROUTINE_ADP_
activated platelets		100.0%	35.3%		34.5%	11.6%	0.698	0.487	0.039	0.320	
all platelets		100.0%	51.8%		66.8%	31.1%	0.826	0.681	0.003	0.228	

Set 1 of variables included: the presence of GPIIIa on the platelet surface, the expression of GPIIb/IIIa complex in resting/activated platelets and the expression of P-selectin in resting/activated platelets; set 2 of variables included: MAX_ADP_, ROUTINE_ADP_, *R*_ADP_/E, netROUTINE_ADP_, MAX_ADP_/*E*, (*R*_ADP_ − *R*)/*R* × 100 and ET capacity. When analyzing the associations separately for resting and ADP-activated platelets (1, 3 and 10 µM ADP), we used resampling with replacement (bootstrap-boosted analysis) adjusted for the overall sample size (*n* = 36). Rho^2^ is a squared canonical correlation coefficient (canonical determination) which is relevant to variance between canonical variables. Total redundancy is relevant to the variance between the canonical variable for a given set and the variables of another set; it shows the representation of a given canonical variable (the first set: activation/reactivity) by the explanatory variables of the other set (mitochondrial respiration). Extracted variance is the variance between a given canonical variable and its constitutive variables; the extracted variance tells us on how far a particular variable is representative of another set of variables (i.e., how redundant the variables of one set are for those of another set). Wilks’ lambda reflects the contribution of one set to explaining the variance of another set; a lower Wilks’ lambda value indicates a higher contribution.

## Data Availability

All data are at the authors’ disposal. The data presented in this study are available on request from the corresponding author. The data are not publicly available due to restrictions applying to donors’ data.

## Supplementary Materials

The following are available online at https://www.mdpi.com/article/10.3390/ijms22179332/s1, Table S1. Biochemical, blood morphology and medical characteristics of participants of the study. Table S2. Platelet P-selectin expression, platelet count and viability of platelet suspensions prepared in autologous plasma or in PBS measured after 0–3 h storage time. Figure S1. The sequential discrete contour line plot representing the “heatmap” of the associations between surface membrane P-selectin expression and mitochondrial respiration parameters and ROX for blood platelets suspended in autologous plasma obtained from blood collected from healthy donors on various anticoagulants. Figure S2. Activation of blood platelets in response to ADP stimulation. Figure S3. The “heatmap” representing the associations between variables describing blood platelet activation and mitochondrial respiration markers for blood platelets from healthy donors incubated with increasing concentrations of ADP.

## References

[1] Fisar Z., Jirak R., Zverova M., Setnicka V., Habartova L., Hroudova J., Vanickova Z., Raboch J. (2019). Plasma amyloid beta levels and platelet mitochondrial respiration in patients with Alzheimer’s disease. Clin. Biochem..

[2] Cardenes N., Corey C., Geary L., Jain S., Zharikov S., Barge S., Novelli E.M., Shiva S. (2014). Platelet bioenergetic screen in sickle cell patients reveals mitochondrial complex V inhibition, which contributes to platelet activation. Blood.

[3] Protti A., Fortunato F., Artoni A., Lecchi A., Motta G., Mistraletti G., Novembrino C., Comi G.P., Gattinoni L. (2015). Platelet mitochondrial dysfunction in critically ill patients: Comparison between sepsis and cardiogenic shock. Crit. Care.

[4] Xu W., Cardenes N., Corey C., Erzurum S.C., Shiva S. (2015). Platelets from Asthmatic Individuals Show Less Reliance on Glycolysis. PLoS ONE.

[5] Karmakar T., Mallick S.K., Chakraborty A., Maiti A., Chowdhury S., Bhattacharyya M. (2015). Signature biomarkers in diabetes mellitus and associated cardiovascular diseases. Clin. Hemorheol. Microcirc..

[6] Yamakawa K., Ogura H., Koh T., Ogawa Y., Matsumoto N., Kuwagata Y., Shimazu T. (2013). Platelet mitochondrial membrane potential correlates with severity in patients with systemic inflammatory response syndrome. J. Trauma Acute Care Surg..

[7] Sjovall F., Morota S., Hansson M.J., Friberg H., Gnaiger E., Elmer E. (2010). Temporal increase of platelet mitochondrial respiration is negatively associated with clinical outcome in patients with sepsis. Crit. Care.

[8] Boudreau L.H., Duchez A.C., Cloutier N., Soulet D., Martin N., Bollinger J., Pare A., Rousseau M., Naika G.S., Levesque T. (2014). Platelets release mitochondria serving as substrate for bactericidal group IIA-secreted phospholipase A2 to promote inflammation. Blood.

[9] Marcoux G., Duchez A.C., Rousseau M., Levesque T., Boudreau L.H., Thibault L., Boilard E. (2017). Microparticle and mitochondrial release during extended storage of different types of platelet concentrates. Platelets.

[10] Ahnadi C.E., Sabrinah Chapman E., Lepine M., Okrongly D., Pujol-Moix N., Hernandez A., Boughrassa F., Grant A.M. (2003). Assessment of platelet activation in several different anticoagulants by the Advia 120 Hematology System, fluorescence flow cytometry, and electron microscopy. Thromb. Haemost..

[11] Soderstrom A.C., Nybo M., Nielsen C., Vinholt P.J. (2016). The effect of centrifugation speed and time on pre-analytical platelet activation. Clin. Chem. Lab. Med..

[12] Maurer-Spurej E., Pfeiler G., Maurer N., Lindner H., Glatter O., Devine D.V. (2001). Room temperature activates human blood platelets. Lab. Investig. A J. Tech. Methods Pathol..

[13] Akkerman J.W., Holmsen H. (1981). Interrelationships among platelet responses: Studies on the burst in proton liberation, lactate production, and oxygen uptake during platelet aggregation and Ca^2+^ secretion. Blood.

[14] Barile C.J., Herrmann P.C., Tyvoll D.A., Collman J.P., Decreau R.A., Bull B.S. (2012). Inhibiting platelet-stimulated blood coagulation by inhibition of mitochondrial respiration. Proc. Natl. Acad. Sci. USA.

[15] Tomasiak M., Stelmach H., Rusak T., Wysocka J. (2004). Nitric oxide and platelet energy metabolism. Acta Biochim. Pol..

[16] Petrus A.T., Lighezan D.L., Danila M.D., Duicu O.M., Sturza A., Muntean D.M., Ionita I. (2019). Assessment of platelet respiration as emerging biomarker of disease. Physiol. Res..

[17] Golebiewska E.M., Poole A.W. (2015). Platelet secretion: From haemostasis to wound healing and beyond. Blood Rev..

[18] Siewiera K., Kassassir H., Talar M., Wieteska L., Watala C. (2016). Higher mitochondrial potential and elevated mitochondrial respiration are associated with excessive activation of blood platelets in diabetic rats. Life Sci..

[19] Ehinger J.K., Morota S., Hansson M.J., Paul G., Elmer E. (2016). Mitochondrial Respiratory Function in Peripheral Blood Cells from Huntington’s Disease Patients. Mov. Disord. Clin. Pract..

[20] Puskarich M.A., Kline J.A., Watts J.A., Shirey K., Hosler J., Jones A.E. (2016). Early alterations in platelet mitochondrial function are associated with survival and organ failure in patients with septic shock. J. Crit. Care.

[21] Golanski J., Pietrucha T., Baj Z., Greger J., Watala C. (1996). Molecular insights into the anticoagulant-induced spontaneous activation of platelets in whole blood-various anticoagulants are not equal. Thromb. Res..

[22] Ritchie J.L., Alexander H.D., Rea I.M. (2000). Flow cytometry analysis of platelet P-selectin expression in whole blood--methodological considerations. Clin. Lab. Haematol..

[23] Gatterer H., Menz V., Salazar-Martinez E., Sumbalova Z., Garcia-Souza L.F., Velika B., Gnaiger E., Burtscher M. (2018). Exercise Performance, Muscle Oxygen Extraction and Blood Cell Mitochondrial Respiration after Repeated-Sprint and Sprint Interval Training in Hypoxia: A Pilot Study. J. Sports Sci. Med..

[24] Karlsson M., Ehinger J.K., Piel S., Sjovall F., Henriksnas J., Hoglund U., Hansson M.J., Elmer E. (2016). Changes in energy metabolism due to acute rotenone-induced mitochondrial complex I dysfunction—An in vivo large animal model. Mitochondrion.

[25] Vevera J., Fisar Z., Nekovarova T., Vrablik M., Zlatohlavek L., Hroudova J., Singh N., Raboch J., Vales K. (2016). Statin-induced changes in mitochondrial respiration in blood platelets in rats and human with dyslipidemia. Physiol. Res..

[26] Malinow A.M., Schuh R.A., Alyamani O., Kim J., Bharadwaj S., Crimmins S.D., Galey J.L., Fiskum G., Polster B.M. (2018). Platelets in preeclamptic pregnancies fail to exhibit the decrease in mitochondrial oxygen consumption rate seen in normal pregnancies. Biosci. Rep..

[27] Jang D.H., Khatri U.G., Mudan A., Love J.S., Owiredu S., Eckmann D.M. (2018). Translational Application of Measuring Mitochondrial Functions in Blood Cells Obtained from Patients with Acute Poisoning. J. Med. Toxicol. Off. J. Am. Coll. Med. Toxicol..

[28] Kramer P.A., Chacko B.K., Ravi S., Johnson M.S., Mitchell T., Darley-Usmar V.M. (2014). Bioenergetics and the oxidative burst: Protocols for the isolation and evaluation of human leukocytes and platelets. J. Vis. Exp..

[29] Vagdatli E., Gounari E., Lazaridou E., Katsibourlia E., Tsikopoulou F., Labrianou I. (2010). Platelet distribution width: A simple, practical and specific marker of activation of coagulation. Hippokratia.

[30] Hechler B., Dupuis A., Mangin P.H., Gachet C. (2019). Platelet preparation for function testing in the laboratory and clinic: Historical and practical aspects. Res. Pract. Thromb. Haemost..

[31] Sjovall F., Ehinger J.K., Marelsson S.E., Morota S., Frostner E.A., Uchino H., Lundgren J., Arnbjornsson E., Hansson M.J., Fellman V. (2013). Mitochondrial respiration in human viable platelets--methodology and influence of gender, age and storage. Mitochondrion.

[32] Wollenman L.C., Vander Ploeg M.R., Miller M.L., Zhang Y., Bazil J.N. (2017). The effect of respiration buffer composition on mitochondrial metabolism and function. PLoS ONE.

[33] Hayashi T., Tanaka S., Hori Y., Hirayama F., Sato E.F., Inoue M. (2011). Role of mitochondria in the maintenance of platelet function during in vitro storage. Transfus. Med..

[34] Perales Villarroel J.P., Figueredo R., Guan Y., Tomaiuolo M., Karamercan M.A., Welsh J., Selak M.A., Becker L.B., Sims C. (2013). Increased platelet storage time is associated with mitochondrial dysfunction and impaired platelet function. J. Surg. Res..

[35] Sowton A.P., Millington-Burgess S.L., Murray A.J., Harper M.T. (2018). Rapid kinetics of changes in oxygen consumption rate in thrombin-stimulated platelets measured by high-resolution respirometry. Biochem. Biophys. Res. Commun..

[36] Przygodzki T., Luzak B., Kassassir H., Mnich E., Boncler M., Siewiera K., Kosmalski M., Szymanski J., Watala C. (2020). Diabetes and Hyperglycemia Affect Platelet GPIIIa Expression. Effects on Adhesion Potential of Blood Platelets from Diabetic Patients under In Vitro Flow Conditions. Int. J. Mol. Sci..

[37] Rywaniak J., Luzak B., Podsedek A., Dudzinska D., Rozalski M., Watala C. (2015). Comparison of cytotoxic and anti-platelet activities of polyphenolic extracts from Arnica montana flowers and Juglans regia husks. Platelets.

[38] Gnaiger E. (2008). Polarographic Oxygen Sensors, the Oxygraph, and High-Resolution Respirometry to Assess Mitochondrial Function. Drug-Induc. Mitochondrial Dysfunct..

[39] Gnaiger E., Renner-Sattler K. (2014). High-resolution respirometry and phosphorylation control protocol with intact cells: ROUTINE, LEAK, ETS, ROX. Mitochondrial Physiol. Netw..

