# Supplementary material for: Sample Preparation as a Critical Aspect of Blood Platelet Mitochondrial Respiration Measurements—The Impact of Platelet Activation on Mitochondrial Respiration
Source: Int J Mol Sci. 2021 Aug 28;22(17):9332. doi: 10.3390/ijms22179332 (PMC8430930; doi:10.3390/ijms22179332)
Supplement: Supplementary file 1 [file ijms-22-09332-s001.zip › Supplementary materials_implemented changes_20210815.pdf]

Table S1. Biochemical, blood morphology and medical characteristics of participants of the study

Blood morphology and biochemical parameters	women (22)	men (7)	together (29)	Statistical significance	Reference ranges women	Reference ranges men
WBC ($10^3/mm^3$)	5.76; 4.85-6.48	5.44; 4.80-6.05	5.69; 4.81-6.28	n.s.	3.98-10.04	4.23-9.07
RBC ($10^6/mm^3$)	4.55 ± 0.31	5.21 ± 0.26	4.71 ± 0.42	*P*<0.0001	3.93-5.22	4.63-6.08
HGB (g/dl)	13.1 ± 1.1	15.2 ± 0.7	13.6 ± 1.4	*P* <0.0001	11.2-15.7	13.7-17.5
HCT (%)	39.5 ± 2.7	45.1 ± 2.0	40.9 ± 3.5	*P* <0.0001	34.1-44.9	40.1-51.0
PLT ($10^3/mm^3$)	248; 208-291	224; 204-257	239; 205-286	n.s.	182-369	163-337
MPV (μm^3)	11.1; 10.4-11.7	10.8; 10.3-11.4	11.0; 10.4-11.6	n.s.	9.4-12.5	9.4-12.6
PCT (%)	0.269; 0.237-0.317	0.247; 0.228-0.269	0.259; 0.234-0.309	n.s.	0.17-0.38	0.16-0.35
PDW (fl)	13.1; 11.8-14.4	13.1; 11.8-14.1	13.1; 11.7-14.4	n.s.	9.8-16.2	9.8-16.1
Lym ($10^3/mm^3$)	1.97 ± 0.52	1.99 ± 0.39	1.97 ± 0.50	n.s.	1.18-3.74	1.32-3.57
Mon ($10^3/mm^3$)	0.496; 0.420-0.615	0.531; 0.477-0.634	0.500; 0.434-0.624	n.s.	0.2-0.8	0.2-0.8
Neu ($10^3/mm^3$)	3.02; 2.52-3.50	2.83; 2.48-3.26	2.97; 2.48-3.46	n.s.	1.56-6.13	1.78-5.38
Eos (10^3/mm3)	0.124; 0.081-0.186	0.189; 0.127-0.294	0.134; 0.084-0.208	n.s.	0.04-0.36	0.04-0.54
Bas ($10^3/mm^3$)	0.038 ± 0.019	0.033 ± 0.013	0.037 ± 0.018	n.s.	0.01-0.08	0.01-0.08
total cholesterol (mmol/l)	4.49; 4.07-4.96	4.58; 4.20-5.13	4.50; 4.08-5.00	n.s.	3.0-5.0	3.0-5.0
triglycerides (mmol/l)	0.78; 0.63-0.99	0.92; 0.75-1.10	0.81; 0.64-1.03	n.s.	<1.7	<1.7
HDL cholesterol (mmol/l)	1.60 ± 0.32	1.43 ± 0.29	1.56 ± 0.33	*P*=0.091*	>1.2	>1.0
LDL cholesterol (mmol/l)	2.52; 2.13-2.83	2.73; 2.39-3.24	2.56; 2.18-2.91	n.s.	<3.0	<3.0
glucose (mmol/l)	4.96; 4.78-5.12	5.07; 4.91-5.29	4.98; 4.80-5.17	n.s.	4.1-5.5	4.1-5.5
uric acid (µmol/l)	260 ± 49	325 ± 65	275 ± 61	*P*=0,015	155-357	208-428
creatinine (µmol/l)	64.9 ± 7.7	84.4 ± 8.9	69.6 ± 11.7	*P* <0.0001	49.0-90.0	64.0-104.0
fibrinogen (mg/dl)	226; 205-251	229; 211-263	225; 206-254	n.s.	150-450	150-450
hsCRP (mg/l)	1.61; 0.85-2.52	0.51; 0.27-1.22	1.19; 0.66-2.37	*P*=0,018	0.0-5.0	0.0-5.0
D-dimer (mg FEU/l)	0.222; 0.152-0.321	0.186; 0.131-0.313	0.212; 0.136-0.316	n.s.	0.1-0.45	0.1-0.45
aPTT ratio	1.008 ± 0.116	1.008 ± 0.112	1.008 ± 0.117	n.s.	0.8-1.2	0.8-1.2
INR (PT)	1.005; 0.968-1.035	0.982; 0.958-1.020	0.999; 0.962-1.035	n.s.	0.8-1.2	0.8-1.2
TT (sec)	17.0 ± 0.6	17.5 ± 0.6	17.1 ± 0.7	*P*=0.047*	14-19	14-19

Variables are presented as means ± SD or medians with interquartile ranges. The two-sided or one-sided (*) a posteriori significance of differences between women and men is given, as calculated with the bootstrap-boosted Student’s t test on the Box-Cox-transformed data. Reference ranges of measured parameters, depending on gender of blood donors, presented in the last two columns, were determined with the automated Sysmex X series counter for blood morphology and Beckman Coulter AU series analyzer for other measured parameters

Abbreviations used: aPTT ratio, activated partial thromboplastin time, ratio; Bas, number of basophils; BMI, body mass index; Eos, number of eosinophils; FEU, fibrinogen equivalent unit, in D-dimer; HCT, haematocrit; HDL, high density lipoprotein cholesterol; HGB, concentration of haemoglobin; hsCRP, high-sensitivity C-reactve protein; INR, international normalized ratio for prothrombin time; LDL, low density lipoprotein cholesterol; Lym, number of lymphocytes; Mon, number of monocytes; MPV, mean platelet volume; Neu, number of neutrophils; n.s., considered ‘non-significant’ with a posteriori $P > 0.1$; PCT, plateletcrit; PDW, platelet distribution width; PLT, platelet count; RBC, red blood cell count; TT, thrombin time; WBC, white blood cell count.

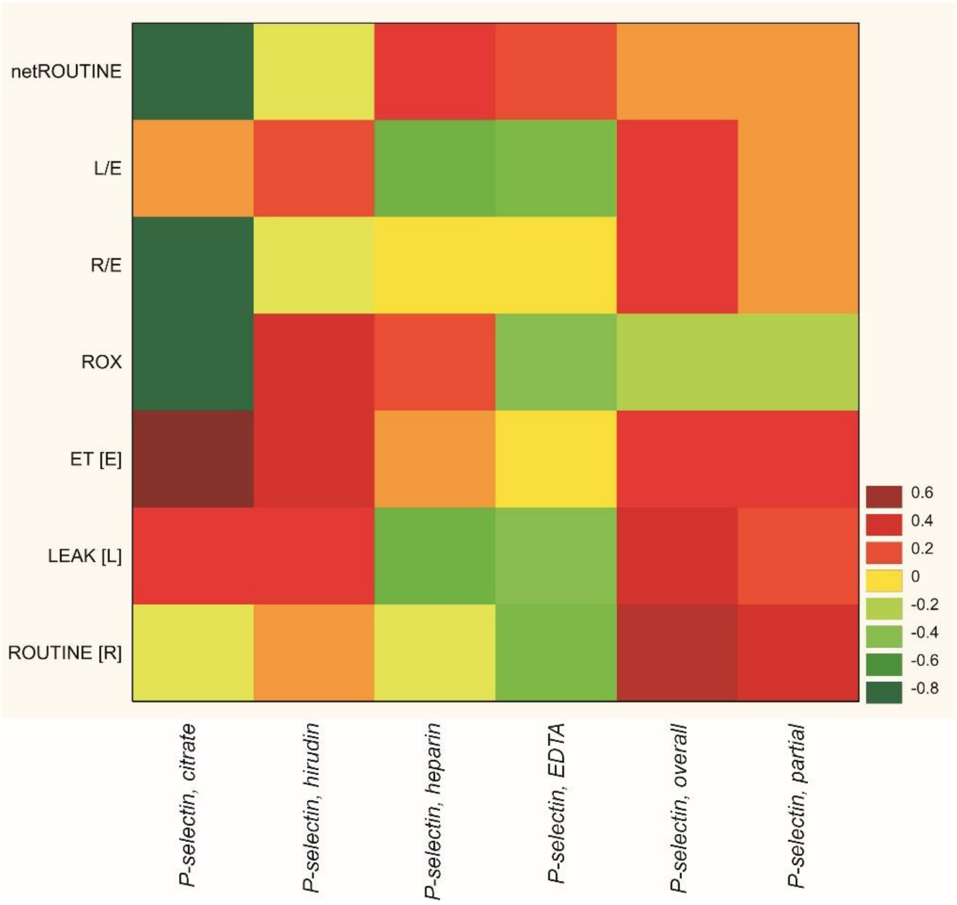

Figure S1. The sequential discrete contour line plot representing the "heatmap" of the associations between surface membrane P-selectin expression and mitochondrial respiration parameters and ROX for blood platelets suspended in autologous plasma obtained from blood collected from healthy donors on various anticoagulants.

The "heatmap" constructed with the use of the bootstrap-boosted overall Spearman correlation coefficients (RS) and the bootstrap-boosted partial Spearman correlation coefficients adjusted for anticoagulant type; the |RS| values > 0.310 remained significant with at least P< 0.05 (for more details see the legend to Table 2 in paragraph 3.1 were numerical values are presented).

Table S2. Platelet P-selectin expression, platelet count and viability of platelet suspensions prepared in autologous plasma or in PBS measured after 0-3 h storage time.

**Variable**		**0h (freshly prepared)**	**1h**	**2h**	**3h**	***Significance***
P-selectin [%]	plasma	7.3; 4.2-10.0	6.8; 5.2-11.8	12.2; 9.3-14.7	15.5 12.2-17.0	*P<0.01 for the effect of time<br>*P<0.01 for the effect of time in plasma<br>*ns. for the effect of time in PBS<br>#for plasma:<br>P<0.05 t1 *vs.* t3; P<0.01 t0 *vs.* t3,<br>ns. t0 *vs.* t1, t0 *vs.* t2, t1 *vs.* t2, t2 *vs.* t3
	PBS	13.6; 9.3–26.7	20,8; 12.2-28.9	17.4; 11.7-23.5	22.6; 15.2-33.0	
PLT count [x10^8]	plasma	21.15; 20.38-23.36	19.80; 19.00-20.83	19.70; 18.60-20.90	20.90; 19.25-23.18	*ns. for the effect of time<br>*ns. for the effect of medium
	PBS	20.65; 19.36-22.11	19.00; 17.50–20.10	19.50; 15.93-21.25	19.60; 15.93-20.13	
Calcein-negative (death) cells [%]	plasma	3.0; 0.2 -10.7	2.0; 0.5–13.0	2.4; 0.7-2.6	3.0; 2.2-9.0	*ns. for the effect of time<br>*ns. for the effect of medium
	PBS	2.6; 0.1-7.8	3.3; 0.5-14.5	1.3; 0.3-0.19.2	4.9; 0.5-8.7	

Data presented as medians and IQR; n=12. Significance of differences were estimated with (*) Friedman's ANOVA (P-selectin expression, PLT count) and (#) the *post-hoc* Dunn's multiple comparison test.

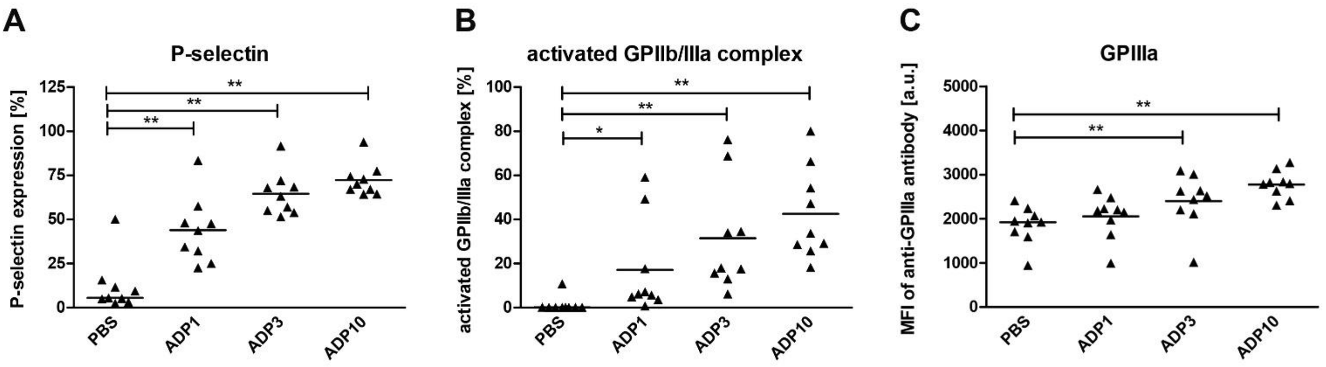

Figure S2. Activation of blood platelets in response to ADP stimulation.

Data presented as individual data points with medians (horizontal lines); n=9 for percentage of P-selectin-positive platelets (A), GPIIb/IIIa (activated complex)-positive platelets (B) or MFI of GPIIIa-bound antibodies (C) in resting and ADP-activated platelets. Three ADP concentrations (1 μM, 2 μM, 10 μM) were tested compared to control (PBS). Significance of differences, as estimated with the ANOVA for repeated measures, and the *post-hoc* Dunnett's test, was: **P*<0.05, ***P*<0.001.

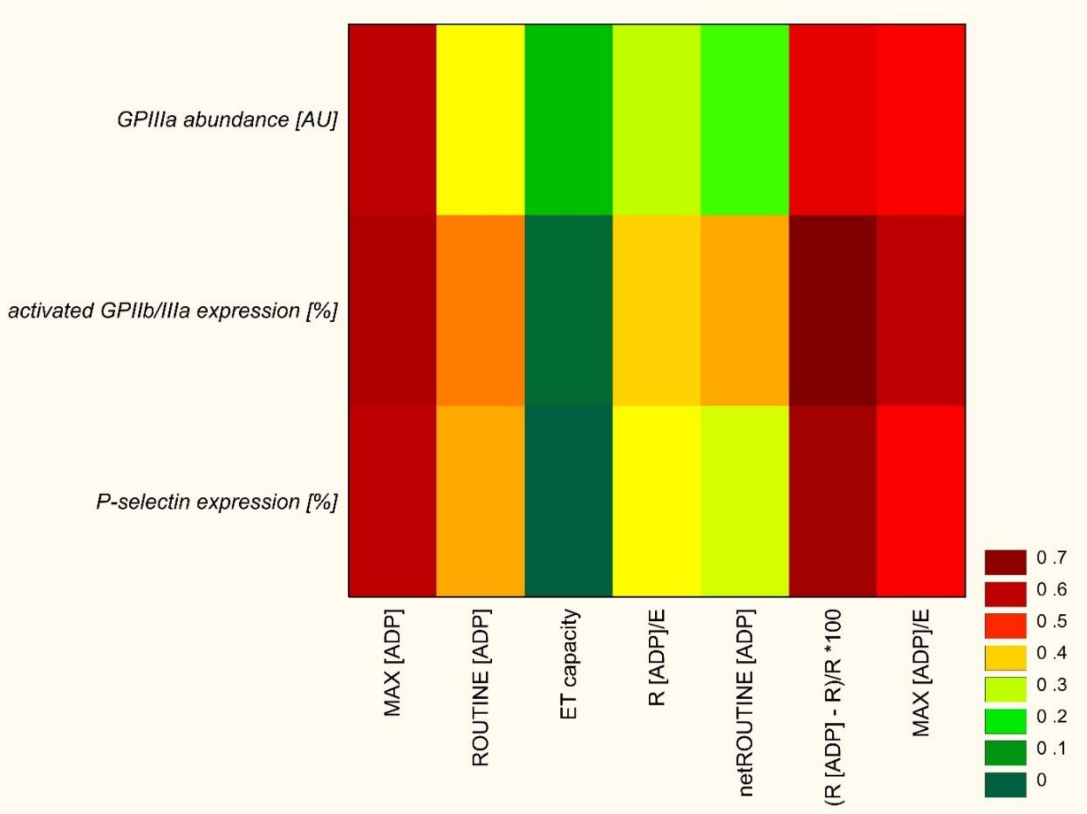

Figure S3. The "heatmap" representing the associations between variables describing blood platelet activation and mitochondrial respiration markers for blood platelets from healthy donors incubated with increasing concentrations of ADP.

Blood was collected on sodium citrate. Isolated platelets were resuspended in plasma/MiR05 combination. Details in Study design, subsection d. The "heatmap" constructed with the use of a sequential discrete contour line plot based on the bootstrap-boosted overall Spearman correlation coefficients (RS); the ǀRSǀ values > 0.300 remained significant with at least P<0.05 (for more details see the legend to Table 5 in in paragraph 3.4 were numerical values were presented).
